# The Predictive Role of NLR and PLR in Outcome and Patency of Lower Limb Revascularization in Patients with Femoropopliteal Disease

**DOI:** 10.3390/jcm11092620

**Published:** 2022-05-06

**Authors:** Eliza Russu, Adrian Vasile Mureșan, Emil Marian Arbănași, Réka Kaller, Ioan Hosu, Septimiu Voidăzan, Eliza Mihaela Arbănași, Cătălin Mircea Coșarcă

**Affiliations:** 1 Clinic of Vascular Surgery, Mureș County Emergency Hospital, 540136 Targu Mures, Romania; eliza.russu@umfst.ro (E.R.); adrian.muresan@umfst.ro (A.V.M.); catalin.cosarca@umfst.ro (C.M.C.); 2 Department of Surgery, George Emil Palade University of Medicine, Pharmacy, Science, and Technology of Targu Mures, 540139 Targu Mures, Romania; 3 Department of Nephrology, Mureș County Emergency Hospital, 540136 Targu Mures, Romania; ioan.hosu@umfst.ro; 4 Department of Epidemiology, George Emil Palade University of Medicine, Pharmacy, Science, and Technology of Targu Mures, 540139 Targu Mures, Romania; septimiu.voidazan@umfst.ro (S.V.); arbanasi.eliza@gmail.com (E.M.A.)

**Keywords:** vascular surgery, NLR, femoropopliteal disease, revascularization, primary patency

## Abstract

Background: Peripheral arterial disease (PAD) changes the arterial structure and function, and is the most common manifestation of the atherosclerotic process, except for the coronary and cerebral arterial systems. Inflammation is well known to have a role in the progression of atherosclerosis and, by extension, in PAD. Among the recently studied markers in the literature, we list the neutrophil–lymphocyte ratio (NLR) and platelet–lymphocyte ratio (PLR). This study aims to analyze the preoperative role of NLR and PLR in the medium-term outcome of patients surgically revascularized for femoropopliteal disease. Methods: A retrospective study included patients admitted to the Vascular Surgery Clinic of the County Emergency Clinical Hospital of Târgu-Mureș, Romania, between January 2017 and December 2019, diagnosed with femoropopliteal disease and having presented an indication for surgical revascularization. The patients included in the study were classified according to the 12 months primary patency in two groups: “patency” and “nonpatency”. Results: Depending on the Rutherford classification (RC), there was a higher incidence of stages II and III in the patency group and a higher incidence of stage V in the nonpatency group. Depending on the optimal cut-off value according to ROC for the 12 months primary patency, obtained from Youden’s index (3.95 for NLR (82.6% sensitivity and 89.9% specificity), and 142.13 for PLR (79.1% sensitivity and 82.6% specificity)), in all high-NLR and high-PLR groups, there was a higher incidence of all adverse outcomes. Moreover, a multivariate analysis showed that a high baseline value for NLR and PLR was an independent predictor of all outcomes for all recruited patients. Furthermore, for all hospitalized patients, RC 5 was an independent predictor of poor prognosis. Conclusions: Our findings establish that a high value of preoperative NLR and PLR determined at hospital admission is strongly predictive of primary patency failure (12 months after revascularization). Additionally, elevated ratio values are an independent predictor for a higher amputation rate and death for all patients enrolled in the study, except for mortality in RC 2, and both amputation and mortality in RC 5.

## 1. Introduction

Peripheral arterial disease (PAD) changes the arterial structure and function, and is the most common manifestation of the atherosclerotic process, except for the coronary and cerebral arterial systems [1,2,3]. Peripheral arterial disease (PAD) has increased globally over the past 20 years, from 1229 patients per 100,000 people in 1990 to 1466 patients per 100,000 people in 2019 [4].

The management of patients diagnosed with PAD is a challenge for specialists. The treatment varies from antithrombotic and anticoagulant medication with the control of risk factors in asymptomatic patients to endovascular or surgical treatment in symptomatic patients [5].

Approximately 20% of patients with PAD have symptoms at the time of diagnosis. This symptomatology ranges from muscle discomfort and intermittent claudication to resting pain with functional impotence, and in the last stage of the Rutherford classification, the appearance of trophic disorders [6,7,8]. Depending on the symptoms and the clinical examination, patients with PAD are classified according to the Rutherford classification (RC) in 6 stages, ranging from the absence of symptoms in stage 0, to intermittent claudication in stages 1–3, rest pain in stage 4, and trophic disorders in stages 5–6 [9]. The most common locations of lesions, the femoropopliteal axis and the popliteal–tibial axis, lead to the most rapid progress to critical ischemia of the lower limb (CLI), corresponding to Rutherford stages 4–6 [10].

Inflammation is well known to have a role in the progression of atherosclerosis and, by extension, in PAD [11,12,13]. As a result, developing predictive inflammatory biomarkers in today’s practice is necessary. Among the recently intensively studied markers in the literature, we list the neutrophil–lymphocyte ratio (NLR) and platelet–lymphocyte ratio (PLR), whose independent roles have been confirmed in various fields, such as oncological surgery, cardiology, cardiac surgery, sepsis, and chronic kidney disease [14,15,16,17,18,19,20,21,22,23,24,25]. Regarding vascular surgery, correlations between high NLR and high PLR values and the outcome in patients diagnosed with abdominal aortic aneurysms have been found [26,27,28,29,30,31].

This study aims to analyze the preoperative roles of NLR and PLR in predicting the primary patency of surgically treated patients with femoropopliteal disease, respectively, the preoperative role of NLR and PLR in predicting amputation, and the mortality of patients with femoropopliteal disease.

## 2. Materials and Methods

### 2.1. Study Design

The present study was designed as an observational, analytical, and retrospective cohort study with longitudinal follow-up, and included all patients diagnosed with femoropopliteal disease admitted to the Vascular Surgery Clinic of the County Emergency Clinical Hospital of Târgu-Mureș, Romania, between January 2017 and December 2019, having presented an indication for surgical revascularization. Exclusion criteria were Rutherford stages 1 and 6, patients diagnosed with systemic inflammatory disease, sepsis, recent tumoral status, hematological diseases, personal history of major surgery in the last six months or recent lower extremity surgical revascularization, autoimmune diseases, and other conditions that induce systemic inflammation. The patients included in the study were classified according to the 12 months primary patency in two groups: “patency” and “nonpatency”.

### 2.2. Data Collection

The patient’s demographic data, the type of revascularization, the number of amputations and fatalities, and the primary patency (12 months follow-up) were collected from the hospital’s computerized database. The following comorbidities were extracted from the medical history: arterial hypertension (AH), atrial fibrillation (AF), chronic heart failure (CHF), ischemic heart disease (IHD), myocardial infarction (MI), type 2 diabetes (T2D), chronic obstructive pulmonary disease (COPD), cerebrovascular accident (CVA), dyslipidemia, tobacco use (history of smoking), and obesity.

### 2.3. Preoperative Workup and Revascularization Technique

Preoperative workup consisted of a physical examination and blood test (glucose level, hemoglobin, hematocrit, neutrophil count, lymphocyte count, monocyte count, and platelet count). A computed tomographic angiography (CTA) was used to properly evaluate the arterial anatomical features of the patients, including the amount of arterial blockage and the number of below-the-knee run-off arteries. Regarding the femoropopliteal axis, we separated the patients into those with superficial femoral artery (SFA) occlusion and those with superficial femoral artery and popliteal artery (SFA + PA) occlusion. For the below-the-knee lesions, we quantified the CTA-objectifiable severe stenoses (>70%) as three categories: <1 affected vessel, 1–2 affected vessels, and 3 affected vessels, respectively.

The NLR and PLR were calculated using the equations below:

$$\mathrm{NLR}=\frac{total\;number\;of\;neutrophils}{total\;number\;of\;lymphocytes}$$

$$\mathrm{PLR}=\frac{total\;number\;of\;platelets}{total\;number\;of\;lymphocytes}$$

Remote endarterectomy of the femoropopliteal axis, above the knee femoropopliteal bypass (AK FP bypass), and below the knee femoropopliteal bypass (BK FP bypass) were the revascularization procedures used. The therapeutic approach was chosen based on the overall biological status of the patients, the degree of arterial occlusion, the grade of atherosclerotic disease, and the expertise of the operating surgeon. The endovascular treatment, unfortunately, was not available in our unit.

### 2.4. Study Outcomes

Primary endpoints were 12 months primary patency, 12 months major amputations rate (including all above the ankle amputations), and 12 months mortality rate. As a second objective, outcomes were stratified for Rutherford classification at hospital admission and both NLR and PLR values at baseline. The Youden index (Youden Index = Sensitivity + Specificity – 1; range from 0 to 1) was used to determine the optimal cut-off values of NLR and PLR using the receiver operating characteristic (ROC) curve analysis.

### 2.5. Ethical Approval

The study was conducted in accordance with the Declaration of Helsinki and approved by the Ethics Committee of Târgu-Mureș Emergency County Hospital, Romania (protocol code 29290, on 10 November 2021). All patients enrolled in the study gave their informed written consent to be included in the present analysis.

### 2.6. Statistical Analysis

Statistical analysis was performed using SPSS for Windows version 28.0.1.0 (SPSS, Inc., Chicago, IL, USA). The associations of NLR and PLR with category variables were assessed using Chi-square test, while differences in continuous variables were analyzed using Student’s *t* test or Mann–Whitney test. The receiver operating characteristic (ROC) curve analysis was used to test the predictive power and determine optimal cut-off values of NLR and PLR. The crude association between elevated NLR, PLR, 12 months primary patency, amputation, and mortality was modeled using Kaplan–Meier curves and compared using the Log Rank test. All tests were two-tailed, and a *p*-value < 0.05 was considered statistically significant.

## 3. Results

The study included 224 patients with femoropopliteal disease who met all the criteria. The mean age of the patients was 69.72 ± 8.34, ranging from 51 to 92 years, with a predominant interest of 74.11% males. The comorbidities with the highest incidence were AH in 186 cases (83.04%), followed by IHD in 181 patients (80.08%), CHF in 142 patients (63.39%), and T2D in 110 patients (49.11%), while the risk factors present were tobacco use (62.95%), hyperlipidemia (59.38%), and obesity (37.5%). The rest of the comorbidities and laboratory data are presented in Table 1.

According to the Rutherford classification, 45 patients (20.09%) were classified in stage two, 69 patients (30.80%) in stage three, 54 patients (24.11%) in stage four, and 56 patients (25%) in stage five. In terms of arterial occlusion, 120 patients had SFA occlusion and 104 had SFA + PA, respectively. Moreover, 28.57% of patients had <1 artery below the knee with severe stenosis, 48.66% had 1–2 arteries, and 22.76% had 3 arteries. Among the operations performed, the AK FP bypass was performed in 139 cases (62.05%), the BK FP bypass in 60 cases (26.79%), and the remote endarterectomy in 25 cases (12.05%). At 12 months postoperatively, in 61.61% of cases, the performed revascularization was patent, 17.86% of patients required an amputation, and 12.05% died (Table 2).

Depending on the 12 months surgery patency, the patients were enrolled in two groups: “patency” and “nonpatency”. Table 3 and Table 4 show the gender distribution, mean age, comorbidities, laboratory data, arterial occlusion, below the knee vessel, Rutherford classification, and type of surgery.

In terms of comorbidities, patients in the nonpatency group had both a higher incidence of AF (*p* = 0.01), CHF (*p* = 0.03), MI (*p* = 0.01), and CKD (*p* = 0.03), as well as a higher incidence of a history of smoking (*p* = 0.002). Regarding the laboratory findings, patients in the second group had lower hemoglobin values (*p* = 0.001), as well as lower values for hematocrit (*p* = 0.0005) and lymphocytes (*p* < 0.0001). There were also high values for glucose (*p* = 0.03), neutrophils (*p* < 0.0001), monocytes (*p* = 0.0003), platelets (*p* < 0.0001), NLR (*p* < 0.0001), and PLR (*p* < 0.0001).

Significant differences were found between the two groups in terms of the Rutherford grading: in the first group, a statistically higher number of patients were admitted with stage two (*p* = 0.03) and three (*p* = 0.02), whereas in the adverse group, there was a higher incidence of stage five (*p* < 0.0001). In terms of the arterial occlusion level and below the knee run-off arteries, in the patency group, we had a higher incidence of SFA occlusion (*p* = 0.0004) and <1 artery with severe stenosis (*p* = 0.0006), while in the nonpatency group, we had a higher incidence of SFA + PA occlusion (*p* = 0.0004) and 3 arteries with severe stenosis (*p* = 0.0006). As per the type of intervention, there was a higher number of patients who benefited from the AK FP bypass (*p* = 0.008) in the first group, and a higher number of patients who benefited from the BK FP bypass (*p* = 0.01) in the second group.

**Figure 1:**
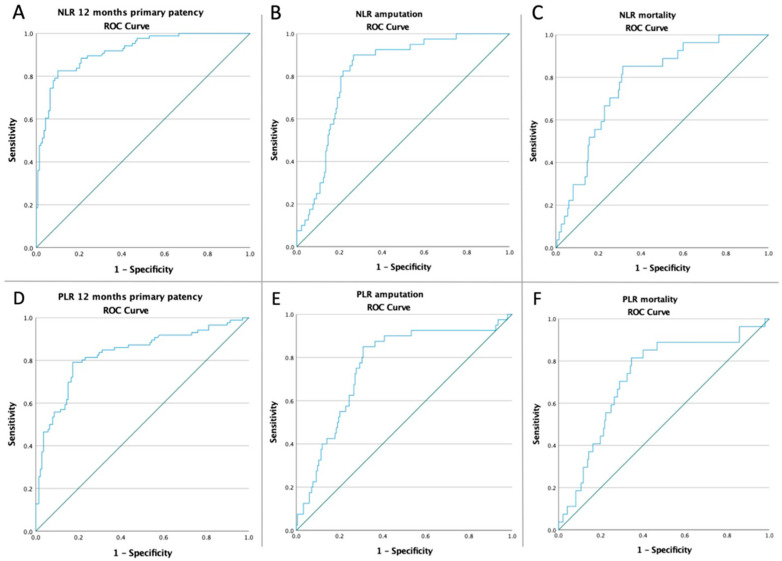
ROC curve analysis (**A**) for NLR concerning 12 months patency in all patients (AUC: 0.915; *p* < 0.001), (**B**) for NLR concerning amputation rate in all patients (AUC: 0.821; *p* < 0.001), (**C**) for NLR concerning the mortality rate in all patients (AUC: 0.777; *p* < 0.001), (**D**) for PLR concerning 12 months patency in all patients (AUC: 0.829; *p* < 0.001), (**E**) for PLR concerning amputation rate in all patients (AUC: 0.760; *p* < 0.001), and (**F**) for PLR concerning the mortality rate in all patients (AUC: 0.722; *p* < 0.001).

The ROC curves for NL, and PLR were created to determine whether the baseline of these biomarkers was predictive of the 12 months primary patency, amputation, and mortality in all patient analyses (Figure 1).

Depending on the optimal cut-off value according to ROC for the 12 months primary patency obtained from Youden’s index (3.95 for NLR (82.6% sensitivity and 89.9% specificity) and 142.13 for PLR (79.1% sensitivity and 82.6% specificity)), the outcomes were further analyzed after dividing the patients into paired groups: low-NLR/high-NLR, and low-PLR/high-PLR. In all high-NLR and high-PLR groups, there was a higher incidence of all adverse outcomes, as seen in Table 5.

**Figure 2:**
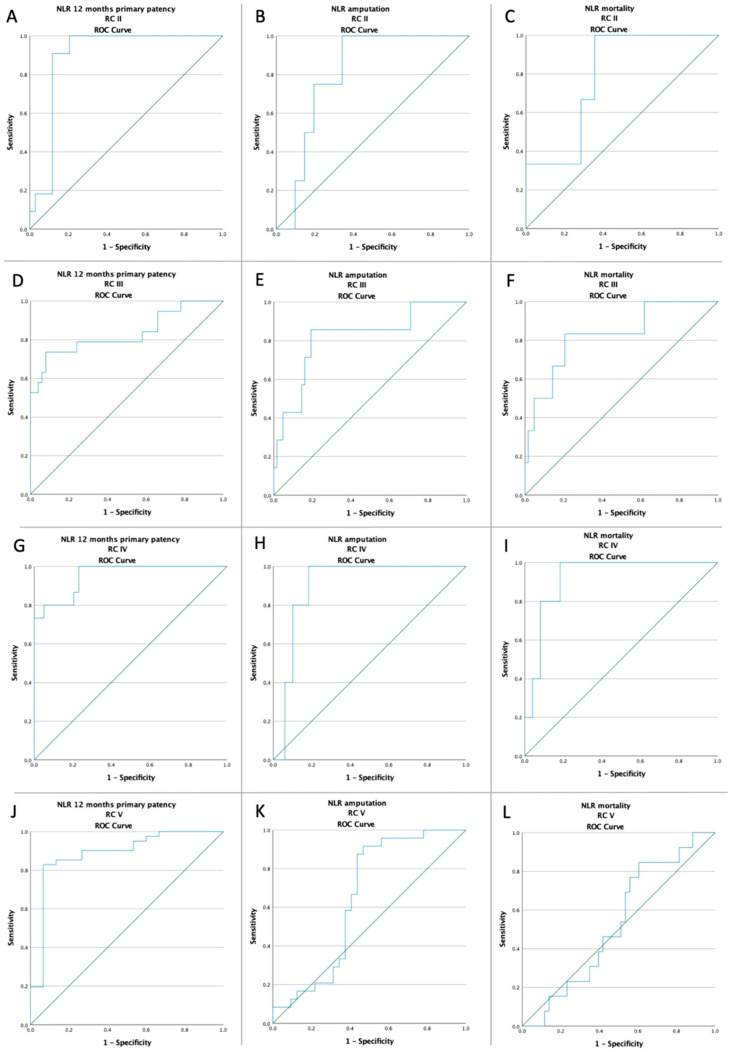
ROC curve analysis (**A**) for NLR with respect to 12 months primary patency in RC 2 patients (AUC: 0.893; *p* < 0.001), (**B**) for NLR with respect to amputation rate in RC 2 patients (AUC: 0.805; *p* = 0.046), (**C**) for NLR with respect to mortality rate in RC 2 patients (AUC: 0.786; *p* = 0.101), (**D**) for NLR with respect to 12 months primary patency in RC 3 patients (AUC: 0.833; *p* < 0.001), (**E**) for NLR with respect to amputation rate in RC 3 patients (AUC: 0.818; *p* = 0.006), (**F**) for NLR with respect to mortality rate in RC 3 patients (AUC: 0.828; *p* = 0.008), (**G**) for NLR with respect to 12 months primary patency in RC 4 patients (AUC: 0.952; *p* < 0.001), (**H**), for NLR with respect to amputation rate in RC 4 patients (AUC: 0.898; *p* = 0.004), (**I**) for NLR with respect to mortality rate in RC 4 patients (AUC: 0.922; *p* = 0.002), (**J**) for NLR with respect to 12 months primary patency in RC 5 patients (AUC: 0.885; *p* < 0.001), (**K**) for NLR with respect to amputation rate in RC 5 patients (AUC: 0.647; *p* = 0.061), and (**L**) for NLR with respect to mortality rate in RC 5 patients (AUC: 0.531; *p* = 0.734); ROC—receiver operating characteristic; NLR—neutrophil-to-lymphocyte ratio; RC—Rutherford classification.

**Figure 3:**
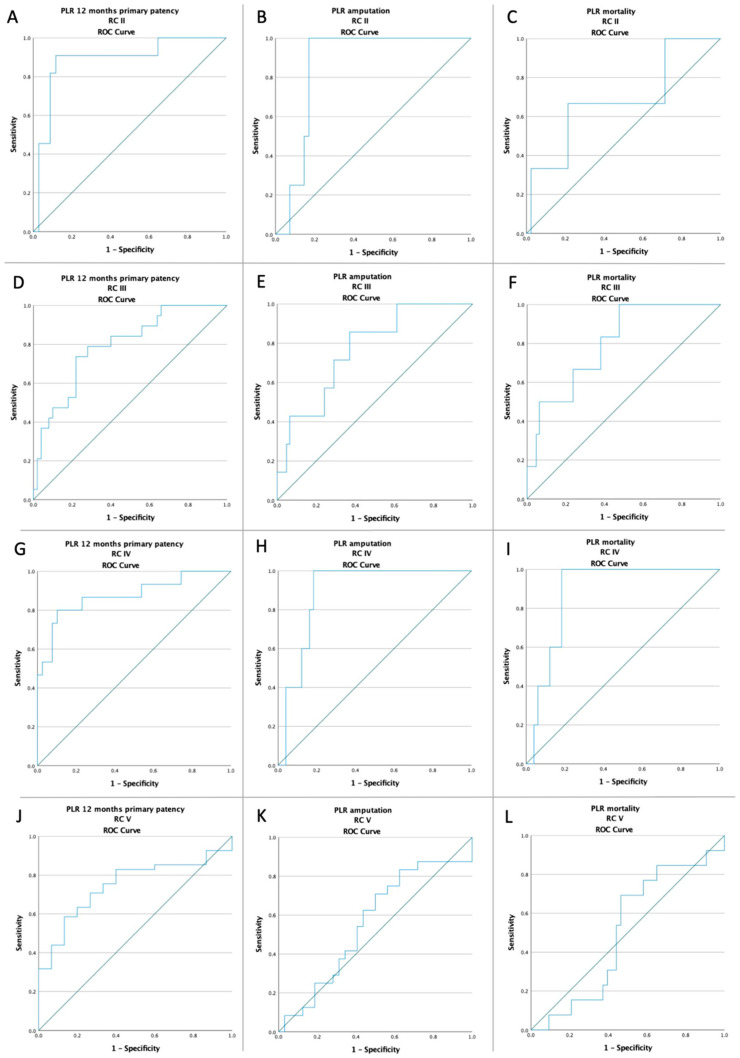
ROC curve analysis (**A**) for PLR with respect to 12 months primary patency in RC 2 patients (AUC: 0.885; *p* < 0.001), (**B**) for PLR with respect to amputation rate in RC 2 patients (AUC: 0.860; *p* = 0.019), (**C**) for PLR with respect to mortality rate in RC 2 patients (AUC: 0.683; *p* = 0.295), (**D**) for PLR with respect to 12 months primary patency in RC 3 patients (AUC: 0.792; *p* < 0.001), (**E**) for PLR with respect to amputation rate in RC 3 patients (AUC: 0.767; *p* = 0.02), (**F**) for PLR with respect to mortality rate in RC 3 patients (AUC: 0.799; *p* = 0.01), (**G**) for PLR with respect to 12 months primary patency in RC 4 patients (AUC: 0.875; *p* < 0.001), (**H**), for PLR with respect to amputation rate in RC 4 patients (AUC: 0.890; *p* = 0.004), (**I**) for PLR with respect to mortality rate in RC 4 patients (AUC: 0.882; *p* = 0.005), (**J**) for PLR with respect to 12 months primary patency in RC 5 patients (AUC: 0.746; *p* = 0.005), (**K**) for PLR with respect to amputation rate in RC 5 patients (AUC: 0.557; *p* = 0.466), and (**L**) for PLR with respect to mortality rate in RC 5 patients (AUC: 0.503; *p* = 0.977); ROC—receiver operating characteristic; PLR—platelet-to-lymphocyte ratio; RC—Rutherford classification.

In the ROC analysis, an NLR value higher than 3.95 and a PLR value higher than 142.13 was strongly associated with all the studied outcomes, except for mortality in RC 2, and both amputation and mortality in RC 5, respectively (Figure 2 and Figure 3).

The multivariate analysis showed that a high baseline value for NLR and PLR was an independent predictor of all outcomes for all recruited patients. Furthermore, for all hospitalized patients, RC V was an independent predictor of poor prognosis (Table 6).

**Figure 4:**
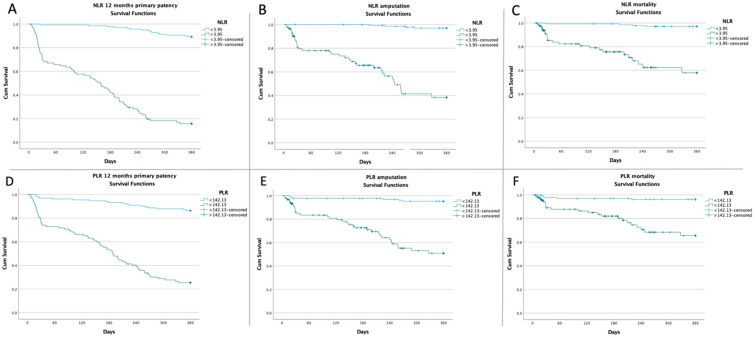
Kaplan–Meier curves showing (**A**) 12 months primary patency probability according to NLR optimal cut-off value (*p* < 0.001; log-rank p), (**B**) amputation probability according to NLR optimal cut-off value (*p* < 0.001; log-rank p), (**C**) mortality probability according to NLR optimal cut-off value (*p* < 0.001; log-rank p), (**D**) 12 months primary patency probability according to PLR optimal cut-off value (p < 0.001; log-rank p), (**E**) amputation probability according to PLR optimal cut-off value (*p* < 0.001; log-rank p), and (**F**) mortality probability according to PLR optimal cut-off value (*p* < 0.001; log-rank p).

The Kaplan–Meier plot for the 12 months primary patency, amputation, and mortality based on the optimal cut-off value of the NLR and PLR for all patients is shown in Figure 4.

## 4. Discussion

The study’s main finding was that preoperative NLR and PLR values had a prognostic role for the primary patency at 12 months in patients with femoropopliteal disease who had a surgical revascularization. Additionally, patients who preoperatively had high NLR and PLR values had a higher incidence of amputation and postrevascularization mortality.

Given the active atherosclerotic inflammatory process, patients with PAD were at a high risk of adverse cardiovascular and other critical events, such as stroke, myocardial infarction, and limb amputation. They had a high mortality rate of up to 26% in 1 year and, respectively, 75% after ten years [32,33,34]. Recent studies have shown the involvement of neutrophils and lymphocytes in atherosclerotic plaque development and implicitly demonstrated their predictive role in the negative evolution of patients with coronary heart disease [35,36,37]. Thus, NLR was based on two parameters that are markers of chronic systemic inflammation with direct involvement in the evolution of atherosclerotic plaque.

NLR and PLR are also inflammatory biomarkers with a prognostic role in the evolution of patients diagnosed with metastatic renal cell carcinoma [38,39,40], the development and stratification of patients with ovarian cancer [41,42,43,44,45], gastric cancer [46,47,48], colorectal cancer [49,50], esophageal cancer [51,52], and breast cancer [53,54].

Spark et al. [55] and Chan et al. [56] found that a preoperative NLR value greater than 5.25 was independently associated with medium-term mortality in patients with CLI and major vascular surgery (58.4% vs. 28.6%; *p* < 0.001) and in patients undergoing infrapopliteal endovascular treatment (39% vs. 17%; *p* = 0.03). Additionally, Bhutta et al. [29] and Gonzalez-Fajardo et al. [57] concluded that the preoperative value of NLR > 5 was associated with long-term mortality in patients with CLI.

In a paper published by Kullar et al., a postoperative NLR value of 5.85 was a prognostic factor for the one-year primary patency in patients with infrainguinal surgical revascularization [58]. Additionally, Taurino et al. demonstrated that an NLR > five at hospitalization in patients with acute lower limb ischemia was an independent prognostic factor for all short-term adverse events [59].

Similar to our study, King et al. published a paper in which they found that a preoperative NLR > 4 was an independent prognostic factor associated with a high mortality rate (*p* = 0.005) and a low amputation-free survival (*p* < 0.0001) in 488 patients following percutaneous interventions on the femoropopliteal segments [60]. Adler et al. analyzed the association of the preoperative value of NLR with adverse outcomes in 92 patients postrevascularization. They demonstrated the predictive role of an NLR value >3.1 regarding mortality (*p* = 0.0001) and major adverse limb events (*p* = 0.049) [61].

In terms of PLR, values above 160 were related to increased amputation rates in patients with CLI, according to a study published by Songur et al. [62]. Demirdal et al. studied 280 patients with diabetic foot infections and found that high NLR and PLR values were related to an increased incidence of amputation [63]. Furthermore, high NLR values were correlated with peripheral arterial disease, and high PLR values were associated with the presence of osteomyelitis. In a paper published by Zhou et al., a PLR value >171 was associated with severe coronary artery stenosis (OR 2.393; 95% CI 1.394–4.108; *p* = 0.002), as well as with a higher rate of major adverse cardiovascular events during the 5-year follow-up (HR, 1.982; 95% CI, 1.329-2.957; *p* = 0.001) [64]. Additionally, Lee et al. highlighted PLR values >137 as an independent predictive factor for all long-term mortality causes (*p* = 0.017) in 514 patients after coronary angiography [65].

According to our results, a preoperative value of NLR > 3.95 was associated with a 12 months primary patency failure (16.47% vs. 89.21%; *p* < 0.0001), with a higher amputation rate (42.35% vs. 2.88%; *p* < 0.0001), and with a higher mortality rate (27.06% vs. 2.88%; *p* < 0.0001). Moreover, a preoperative value of PLR > 142.13 was also associated with a 12 months primary patency failure (26.09% vs. 86.38%; *p* < 0.0001), with a higher amputation rate (36.96% vs. 4.55%; *p* < 0.0001), and with a higher mortality rate (23.91% vs. 3.79%; *p* < 0.0001). Additionally, according to the ROC analysis, depending on the Rutherford classification, a value of NLR > 3.95 and a value of PLR > 142.13 were associated with all significant adverse events, except for mortality in RC 2, and both amputation and mortality in RC 5, as seen in Figure 2 and Figure 3. The plausible explanation is that, regardless of revascularization, these patients had a far too locally advanced affliction to be downstaged or their outcome to be significantly influenced therapeutically. The inflammation residing in the complex soft tissue lesions was biasing the results. The Kaplan–Meier survival plot at 12 months showed a higher rate of surgical revascularization failure in patients with a preoperative NLR value >3.95 (*p* < 0.001; log-rank test) and a preoperative PLR value >142.13 (*p* < 0.001; log-rank test). The difference was also maintained in terms of amputation and mortality, as seen in Figure 4.

Considering the easiness and the almost insignificant cost of calculating the NLR and PLR, these ratios can be used in the preoperative stratification of patients in risk groups, correlated with the Rutherford classification, for the better management of patients and the establishment of predictive hypotheses. Moreover, we considered that NLR and PLR may raise clinical susceptibility towards poor outcomes after revascularization, making them the first line of a predictive set of biochemical surveillance. We recommend calculating them before and after trombendarterectomies and bypasses, as valuable tools in the armamentarium of vascular care.

Despite the results, our study had some limitations. First of all, it was a retrospective study, with a relatively small number of patients, and from a single center, in which the medium-term outcome was monitored. In the future, we recommend conducting a prospective, multicenter study with long-term outcome monitorization and recording of the causes of primary patency failure. Another limitation was the sole recruitment of patients who had benefited from surgical revascularization; therefore, the results could not be extrapolated to patients with an indication for endovascular treatment. In the future, we suggest recording the NLR and PLR values both preoperatively and postoperatively and confirming the prognostic relevance of these values and the difference between the two values regarding the adverse events of postrevascularization patients.

## 5. Conclusions

Our findings concluded that higher preoperative NLR and PLR values determined at hospital admission were strongly predictive of the 12 months primary patency failure. Additionally, elevated values of the ratios were an independent predictor of a higher amputation rate and fatality for all patients enrolled in the study, except for mortality in RC 2 and both amputation and mortality in RC 5. Given the accessibility and low cost of the ratios, they can be considered for preoperative risk group stratification, correlated with the Rutherford classification, for the better management of patients and for developing predictive patterns.

## Figures and Tables

**Table 1:** Demographic, comorbidities, risk factors, and laboratory findings for all patients.

Variables	All Patients n = 224
Age mean ± SD (min-max)	69.72 ± 8.34 (51–92)
Male sex no. (%)	166 (74.11%)
Comorbidities and Risk factors	
AH, no. (%)	186 (83.04%)
IHD, no. (%)	181 (80.80%)
AF, no. (%)	53 (23.66%)
CHF, no. (%)	142 (63.39%)
MI, no. (%)	79 (35.27%)
COPD, no. (%)	56 (25.00%)
T2D, no. (%)	110 (49.11%)
CVA, no. (%)	61 (27.23%)
CVI, no. (%)	50 (22.32%)
CKD, no. (%)	53 (23.66%)
Tobacco, no. (%)	141 (62.95%)
Obesity, no. (%)	84 (37.5%)
Hyperlipidemia, no. (%)	133 (59.38%)
Laboratory data	
Hemoglobin g/dL median (Q1–Q3)	12.36 (10.33–13.92)
Hematocrit % median (Q1–Q3)	37.33 (31.94–41.92)
Glucose mg/dL median (Q1–Q3)	106 (92–134)
Neutrophils ×10^3^/uL median (Q1–Q3)	6.24 (4.77–8.42)
Lymphocytes ×10^3^/uL median (Q1–Q3)	1.90 (1.45–2.62)
Monocyte ×10^3^/uL median (Q1–Q3)	0.61 (0.46–0.79)
PLT ×10^3^/uL median (Q1–Q3)	251.35 (208.32–314.35)
NLR median (Q1–Q3)	2.98 (1.97–5.81)
PLR median (Q1–Q3)	126.21 (94.24–181.99)

AH—arterial hypertension; IHD—ischemic heart disease; AF—atrial fibrillation; CHF—chronic heart failure; MI—myocardial infarction; COPD—chronic obstructive pulmonary disease; T2D—type 2 diabetes; CVA—cerebrovascular accident; PLT—total platelet count.

**Table 2:** Rutherford classification, arterial occlusion, below the knee run-off arteries, type of surgery, and outcome for all patients.

Variables	All Patients n = 224
Rutherford classification	
Stg 2, no. (%)	45 (20.09%)
Stg 3, no. (%)	69 (30.80%)
Stg 4, no. (%)	54 (24.11%)
Stg 5, no. (%)	56 (25.00%)
Arterial occlusion	
SFA, no. (%)	120 (53.57%)
SFA + PA, no. (%)	104 (46.42%)
Below the knee run-off arteries	
<1	64 (28.57%)
1–2	109 (48.66%)
3	51 (22.76%)
Type of surgery	
Remote endarterectomy, no. (%)	25 (11.16%)
AK FP bypass, no. (%)	139 (62.05%)
BK FP bypass, no. (%)	60 (26.79%)
Outcome	
12 months primary patency, no. (%)	138 (61.61%)
Amputation, no. (%)	40 (17.86%)
Death, no. (%)	27 (12.05%)

SFA—superficial femoral artery; PA—popliteal artery; AK—above the knee; BK—below the knee; FP—femoropopliteal.

**Table 3:** Demographic data, comorbidities, risk factors, and laboratory findings of the two subgroups were evaluated according to patency of performed revascularization.

	Patency n = 138	Nonpatency n = 86	*p*-Value (OR; CI 95%)
Age mean ± SD (min–max)	69.56 ± 7.86 (52–92)	69.98 ± 9.10 (51–89)	0.72 ^a^
Male sex no. (%)	105 (76.09%)	61 (70.93%)	0.39 ^b^ (0.76; 0.41–1.40)
Comorbidities and Risk factors			
AH, no. (%)	110 (79.71%)	76 (88.37%)	0.09 ^b^ (1.93; 0.88–4.21)
IHD, no. (%)	107 (77.54%)	74 (86.05%)	0.11 ^b^ (1.78; 0.86–3.70)
AF, no. (%)	25 (18.12%)	28 (32.56%)	0.01 ^b^ (2.18; 1.16–4.07)
CHF, no. (%)	80 (57.97%)	62 (72.09%)	0.03 ^b^ (1.87; 1.04–3.34)
MI, no. (%)	40 (28.99%)	39 (45.35%)	0.01 ^b^ (2.03; 1.15–3.56)
COPD, no. (%)	33 (23.91%)	23 (26.74%)	0.63 ^b^ (1.16; 0.62–2.15)
T2D, no. (%)	66 (47.83%)	44 (51.16%)	0.62 ^b^ (0.87; 0.51–1.49)
CVA, no. (%)	36 (26.09%)	25 (29.07%)	0.62 ^b^ (1.14; 0.66–1.95)
CVI, no. (%)	34 (24.64%)	16 (18.6%)	0.29 ^b^ (0.69; 0.35–1.36)
CKD, no. (%)	26 (18.84%)	27 (30.23%)	0.03 ^b^ (1.97; 1.05–3.67)
Tobacco, no. (%)	76 (55.07%)	65 (75.58%)	0.002 ^b^ (2.52; 1.39–4.58)
Obesity, no. (%)	51 (36.96%)	33 (38.37%)	0.83 ^b^ (1.06; 0.60–1.85)
Hyperlipidemia, no. (%)	81 (58.7%)	52 (60.47%)	0.79 ^b^ (1.07; 0.62–1.86)
Laboratory data			
Hemoglobin g/dL median (Q1–Q3)	12.75 (10.96–14.24)	11.35 (9.72–13.23)	0.001 ^c^
Hematocrit % median (Q1–Q3)	39.26 (33.4–42.97)	34.86 (30.52–40.29)	0.0005 ^c^
Glucose mg/dL median (Q1–Q3)	102.4 (91–126)	116.3 (95.25–140.62)	0.03 ^c^
Neutrophils ×10^3^/uL median (Q1–Q3)	5.24 (4.03–6.58)	8.85 (6.83–11.14)	<0.0001 ^c^
Lymphocytes ×10^3^/uL median (Q1–Q3)	2.25 (1.77–2.96)	1.46 (1.13–1.86)	<0.0001 ^c^
Monocyte ×10^3^/uL median (Q1–Q3)	0.59 (0.44–0.72)	0.70 (0.51–0.94)	0.0003 ^c^
PLT ×10^3^/uL median (Q1–Q3)	238.45 (202.7–293.85)	283.65 (227.75–416.85)	<0.0001 ^c^
NLR median (Q1–Q3)	2.21 (1.66–2.99)	6.40 (4.64–8.83)	<0.0001 ^c^
PLR median (Q1–Q3)	108.02 (82.81–131.55)	191.51 (145.5–273.58)	<0.0001 ^c^

AH—arterial hypertension; IHD—ischemic heart disease; AF—atrial fibrillation; CHF—chronic heart failure; MI—myocardial infarction; COPD—chronic obstructive pulmonary disease; T2D—type 2 diabetes; CVA—cerebrovascular accident; PLT—total platelet count; ^a^, Student’s *t* test; ^b^, chi square test; ^c^, Mann–Whitney test.

**Table 4:** Rutherford classification, arterial occlusion, below the knee run-off arteries, and type of surgery of the two subgroups were evaluated according to patency of performed revascularization.

	Patency n = 138	Nonpatency n = 86	*p*-Value ^b^ (OR; CI 95%)
Rutherford classification			
stg 2, no. (%)	34 (24.64%)	11 (12.79%)	0.03 ^b^ (0.44; 0.21–0.94)
stg 3, no. (%)	50 (36.23%)	19 (22.09%)	0.02 ^b^ (0.49; 0.26–0.92)
stg 4, no. (%)	39 (28.26%)	15 (17.44%)	0.06 ^b^ (0.53; 0.27–1.04)
stg 5, no. (%)	15 (10.87%)	41 (47.67%)	<0.0001 ^b^ (7.47; 3.77–14.279)
Arterial occlusion			
SFA, no. (%)	87 (63.04%)	33 (38.37%)	0.0004 ^b^ (0.36; 0.20–0.63)
SFA + PA, no. (%)	51 (36.96%)	53 (61.63%)	0.0004 ^b^ (2.73; 1.57–4.77)
Below the knee run-off arteries			
<1	51 (36.96%)	13 (15.11%)	0.0006 ^b^ (0.30; 0.15–0.60)
1–2	64 (46.37%)	45 (52.32%)	0.38 ^b^ (1.26; 0.73–2.17)
3	23 (16.67%)	28 (32.57%)	0.0006 ^b^ (2.41; 1.27–455)
Type of surgery			
Remote endarterectomy, no. (%)	14 (10.14%)	11 (12.79%)	0.54 ^b^ (1.29; 0.56–3.00)
AK FP bypass, no. (%)	95 (68.84%)	44 (51.16%)	0.008 ^b^ (0.47; 0.27–0.82)
BK FP bypass, no. (%)	29 (21.01%)	31 (36.05%)	0.01 ^b^ (2.11; 1.16–3.86)

SFA—superficial femoral artery; PA—popliteal artery; AK—above the knee; BK—below the knee; FP—femoropopliteal; ^b^, chi square test.

**Table 5:** Univariate analysis of NLR, PLR, and all adverse event occurrences during the study period for all patients.

NLR = 3.95	12 Months Primary Patency	Amputation	Mortality
LOW-NLR VS. HIGH-NLR	124/139 (89.21%) vs. 14/85 (16.47%) *p* < 0.0001 OR:0.02 CI: (0.01–0.05)	4/139 (2.88%) vs. 36/85 (42.35%) *p* < 0.0001 OR:24.79 CI: (8.39–73.27)	4/139 (2.88%) vs. 23/85 (27.06%) *p* < 0.0001 OR:12.52 CI: (4.15–37.74)
PLR = 142.13	12 Months Primary Patency	Amputation	Mortality
LOW-PLR VS. HIGH-PLR	114/132 (86.38%) vs. 24/92 (26.09%) *p* < 0.0001 OR:0.05 CI: (0.02–0.11)	6/132 (4.55%) vs. 34/92 (36.96%) *p* < 0.0001 OR:12.31 CI: (4.89–30.95)	5/132 (3.79%) vs. 22/92 (23.91%) *p* = 0.0001 OR:7.98 CI: (2.89–22)

**Table 6:** Multivariate analysis on new adverse event occurrences during the entire study period.

	12 Months Primary Patency			Amputation			Mortality		
	OR	95% CI	*p* Value	OR	95% CI	*p* Value	OR	95% CI	*p* Value
AF	2.18	1.16–4.07	0.01	1.73	0.82–3.66	0.15	1.14	0.45–2.88	0.76
MI	1.59	0.90–2.78	0.10	1.45	0.72–2.92	0.29	1.55	0.68–3.50	0.29
CVA	1.16	0.63–2.11	0.62	1.18	0.55–2.51	0.66	1.14	0.47–2.76	0.76
CKD	1.86	0.99–3.49	0.051	1.32	0.60–2.87	0.48	2.17	0.92–5.09	0.07
Tobacco	2.52	1.39–4.58	0.002	3.31	1.39–2.87	0.007	2.24	0.86–5.81	0.09
RC II	0.44	0.21–0.94	0.03	0.38	0.13–1.15	0.46	0.46	0.13–1.60	0.22
RC III	0.49	0.26–0.92	0.02	0.41	0.17–0.99	0.04	0.60	0.23–1.58	0.30
RC IV	0.53	0.27–1.04	0.06	0.39	0.14–1.06	0.06	0.68	0.24–1.91	0.057
RC V	7.47	3.77–14.79	<0.001	7.12	3.40–14.91	<0.001	3.32	1.45–7.60	0.004
high-NLR	41.92	19.13–91.87	<0.001	24.79	8.39–73.27	<0.001	12.52	4.15–37.74	<0.001
high-PLR	17.94	9.08–35.45	<0.001	12.31	4.89–30.95	<0.001	7.98	2.89–22.00	<0.001
